# Treatment of Cognitive Impairment Associated with Schizophrenia Spectrum Disorders: New Evidence, Challenges, and Future Perspectives

**DOI:** 10.3390/brainsci14080791

**Published:** 2024-08-06

**Authors:** Irene Calzavara-Pinton, Gabriele Nibbio, Stefano Barlati, Lorenzo Bertoni, Nicola Necchini, Daniela Zardini, Antonio Baglioni, Stefano Paolini, Laura Poddighe, Viola Bulgari, Jacopo Lisoni, Giacomo Deste, Antonio Vita

**Affiliations:** 1 Department of Mental Health and Addiction Services, ASST Spedali Civili of Brescia, 25123 Brescia, Italy; irene.calzavarapinton@gmail.com (I.C.-P.); laura.poddighe18@gmail.com (L.P.); jacopo.lisoni@gmail.com (J.L.); antonio.vita@unibs.it (A.V.); 2 Department of Clinical and Experimental Sciences, University of Brescia, 25123 Brescia, Italy; gabriele.nibbio@gmail.com (G.N.); l.bertoni005@unibs.it (L.B.); nnecchini@gmail.com (N.N.); dani.zardi@gmail.com (D.Z.); a.baglioni@unibs.it (A.B.); s.paolini@unibs.it (S.P.); viola.bulgari2@gmail.com (V.B.); giacomodeste@mac.com (G.D.); 3 Department of Mental Health, ASST Valcamonica, 25040 Brescia, Italy

**Keywords:** CIAS, cognition, cognitive remediation, physical exercise, evidence-based, psychosocial interventions, schizophrenia

## Abstract

Cognitive impairment associated with schizophrenia (CIAS) represents one of the core features of the disorder and has a significant impact on functional and rehabilitation outcomes of people living with schizophrenia spectrum disorders (SSD). The aim of this critical review is to highlight the most recent evidence on effective treatments available for CIAS, to discuss the current challenges in this field, and to present future perspectives that may help to overcome them. Concerning psychopharmacological approaches, among the most indicated strategies for the management and prevention of CIAS is to favor second-generation antipsychotic medications and avoid long-term and high-dose treatments with anticholinergic medications and benzodiazepines. Moreover, non-pharmacological approaches such as cognitive remediation and physical exercise-based programs represent evidence-based interventions in the treatment of CIAS that have shown reliable evidence of effectiveness on both cognitive and functional outcomes. These treatments, however, are still delivered to people accessing mental health services with a diagnosis of CIAS in an uneven manner, even in high-income countries. Academic and clinical partnership and collaboration, as well as advocacy from service users, families, carers, and stakeholders’ organizations could help to reduce the bench to bedside gap in the treatment of CIAS. Future perspectives include the development of novel pharmacological agents that could be effective in the treatment of CIAS, the implementation of novel technologies such as telemedicine and virtual reality in the delivery of evidence-based interventions to improve accessibility and engagement, and further research in the field of non-invasive brain stimulation.

## 1. Introduction

Schizophrenia spectrum disorders (SSD) are debilitating mental disorders that are often associated with psychosocial functioning impairment and poor real-world outcomes [1,2,3,4,5,6], as well as elevated levels of internalized stigma [7,8,9].

Cognitive impairment represents one of the core features of SSD [10,11,12], and has been considered as such since the earliest conceptualizations of the disorder [13,14]. In fact, it can be observed in the vast majority of diagnosed individuals [15,16,17] from an early age, even before the onset of psychotic symptoms [18,19,20,21], and it involves both neurocognitive [22,23] and social cognition domains [24,25,26].

Cognitive impairment also represents one of the essential determinants of real-world functional impairment in people living with SSD [27,28,29] and a considerable impact on more distal outcomes that are considered as relevant treatment goals for patients such as quality of life [30,31,32] and life engagement [33,34]. Moreover, cognitive impairment appears to play a consistent negative role in the process of recovery of people living with SSD, acting as a barrier in the context of psychiatric rehabilitation of SSD [35,36,37], and is also related to the potential of aggressive behavior [38,39,40,41].

Furthermore, the expression of cognitive impairment in individuals living with SSD can be very heterogeneous: it is often difficult to determine premorbid cognition, therefore making it harder to determine to what extent it is intrinsic to the disorder and to what extent it can be influenced by several aspects such as culture, age, and education [42,43].

Considering the frequency, the relevance, and the impact that cognitive impairment has on the lives of people with SSD, the scientific community is gravitating towards the use of a dedicated term to identify this psychopathological entity, that of cognitive impairment associated with schizophrenia (CIAS) [44,45].

Despite the well-known clinical significance of CIAS, the treatment of CIAS remains complex and several unresolved issues and challenges remain about this topic. For instance, there is no pharmacological agent that has been currently approved specifically for the treatment CIAS [46,47,48,49,50]. In addition, many currently widely used antipsychotic medications (mainly first-generation antipsychotics) have been proved to worsen CIAS due to different molecular mechanisms [50,51]. In addition, many different neurobiological mechanisms have been identified as correlates of CIAS, such as aberrant neural network organization, grey matter volume reduction, altered neuronal functioning and neurotransmission, and excitatory/inhibitory imbalance at a cortical level, therefore making it harder to select specific molecular targets and study new medications [52,53,54]. Furthermore, non-pharmacological treatments like cognitive remediation have been extensively studied but, although effective, they have limitations, such as the need for trained staff and integration with rehabilitation [55].

Despite the importance of CIAS both in a scientific and in a clinical perspective, most people living with SSD do not receive adequate treatment for CIAS in routine clinical practice, even in high-income countries and in contexts with considerable levels of available resources [56,57,58]. This is probably due to the combined effect of different factors; nonetheless, addressing this issue currently represents one of the main goals in the field of SSD treatment.

The aims of the present critical review are to highlight the most recent evidence on effective treatments available for CIAS, to discuss the current challenges in this field, and to present future perspectives that may help to overcome them [50].

Narrative reviews form the basis of medical literature synthesis: unlike scoping reviews, they can consider several different questions at the same time, and unlike systematic reviews they can include the opinions, perspectives, and speculations of experts beside and beyond the evidence reported in primary research. In this context, they can provide a critical assessment of the available evidence and offer valuable insight for future research [59,60,61].

## 2. Treatment of Cognitive Impairment Associated with Schizophrenia

As previously mentioned, effective treatment of CIAS represents one of the main goals in the field of clinical psychiatry when focusing on SSD.

To address CIAS, both pharmacological approaches and psychosocial interventions have been devised and are being currently investigated, showing different degrees of effectiveness.

### 2.1. Pharmacological Treatment

The currently available pharmacological agents do not represent an effective treatment for CIAS. In fact, no molecule has been approved specifically for the treatment of CIAS, and no medication is currently recommended to improve CIAS in any international treatment guideline [46,47,48,49,50].

However, an accurate management of pharmacological treatment could be of considerable help in the management of CIAS by avoiding approaches that have a negative impact on cognitive performance.

The recent European Psychiatric Association guidance dedicated to treatment of CIAS [50] recommends with the highest available grading to prefer second-generation antipsychotics due to their favorable cognitive profile and to switch to a second-generation molecule in patients with CIAS treated with a first-generation antipsychotics.

In fact, second-generation antipsychotics outperformed first-generation ones on cognitive outcomes in different systematic reviews and meta-analyses, while first-generation agents performed worse than the placebo [62,63,64]. No second-generation molecule emerged as superior to the others in systematic assessments [65,66]; recently-developed antipsychotics such as lurasidone, cariprazine, brexpiprazole, and lumateperone have been postulated to have more favorable cognitive effects due to their pharmacodynamic profile [67,68,69,70], but these assumptions need to be more reliably proven by solid research and stronger evidence.

Reducing the anticholinergic burden represents another essential step to optimize pharmacological treatment in order to avoid worsening CIAS [71,72,73,74,75,76,77,78,79]: in this perspective, antipsychotics with a lower anticholinergic burden should be preferred when possible, and, most importantly, long-term and high dose treatments with anticholinergic medications should be avoided.

Benzodiazepines represent another category of medication that could have a negative impact on CIAS [80,81], and again, long-term, high dose treatments should be avoided, particularly in individuals already showing CIAS.

While no molecule has been approved or recommended for CIAS, some medications have shown preliminary evidence of effectiveness [45,82]. Some positive effects on CIAS have been observed with medications that are usually prescribed in the context of stages of neurodegenerative conditions producing cognitive impairment and dementia, such as Alzheimer’s Disease: preliminary evidence of positive effects has been observed with acetylcholinesterase inhibitors such as memantine [83,84,85] and galantamine (which also acts as a positive allosteric modulator of alpha-7 nicotinic acetylcholine receptors) [86]. Preliminary positive effects have been observed also using anti-inflammatory molecules [87,88,89,90], particularly N-acetylcysteine [91] and minocycline [90,92,93].

Promising evidence has also been shown by molecules that are currently being developed and investigated in the treatment of SSD in regulatory trials, such as iclepertin [94,95,96,97], luvadaxistat [98,99,100], and xanomeline–trospium [101,102].

In particular, iclepertin (registered as BI 425809) is a selective glycine transporter 1 inhibitor which is currently being investigated as an add-on treatment to antipsychotic medications, aiming to improve negative symptoms of SSD and CIAS [94]. Preliminary evidence of its effectiveness on CIAS has been reported in a Phase II placebo-controlled randomized trial including 509 participants (NCT02832037), where the two higher-doses (10 and 25 mg) iclepertin groups significantly outperformed placebo considering the MATRICS Consensus Cognitive Battery global cognitive scores as outcome [97].

Luvadaxistat (registered as TAK-831 and NBI-1065844) is a D-amino acid oxidase that is also being investigated as an add-on to antipsychotic medications for the treatment of negative symptoms and CIAS [98]. The results of a randomized, placebo-controlled trial including 256 participants were recently published. No significant positive effects were observed as regard negative symptoms; however, in the lowest dose group (50 mg), luvadaxistat outperformed placebo on both performance-based measures of CIAS such as the Brief Assessment of Cognition in Schizophrenia composite score and interview-based assessments such as the Schizophrenia Cognition Rating Scale score [100].

Xanomeline–trospium (or KarXT) is a combination of muscarinic agonist acting on M1, M4, and M5 receptors (xanomeline) and a peripheral muscarinic receptor antagonist that does not cross the blood–brain barrier, reducing cholinergic adverse effects without hindering the therapeutic effects (trospium) [103]. Xanomeline–trospium is being investigated as a stand-alone antipsychotic medication based on a mechanism of action that does not involve D2 dopamine receptor blocking or modulation, and evidence of its effectiveness on positive and negative symptoms of SSD has been provided by the recently published results of a Phase 3 randomized clinical trial (NCT04659161) including 252 participants [102]. However, the results of a Phase 2 trial (NCT03697252) including 125 participants also reported interesting findings regarding CIAS. The computerized Cogstate Brief Battery was used to measure cognitive performance, and while no significant treatment-related effect was observed in the primary analysis, post hoc analyses conducted by removing outliers showed significant positive effects that were not related to psychotic symptom improvements and that were of moderate effects size in participants starting the trial with significant CIAS (defined as a composite cognitive Z score < −1) [101].

The results of Phase 2 and Phase 3 trials that are currently underway will reveal whether any of these molecules represents the long-awaited pharmacological game-changer in the treatment of CIAS. However, even in the absence of highly impactful pharmacological solutions, an effective and currently available way to treat CIAS is represented by evidence-based psychosocial interventions.

Pharmacological interventions currently under investigation and of interest for CIAS treatment are summarized in Table 1.

### 2.2. Psychosocial Interventions

Psychosocial interventions currently represent the most effective strategy to address CIAS [59,104,105,106].

Cognitive remediation (CR), in particular, is the intervention with the highest available degree of recommendation in the European Psychiatric Association guidance for the treatment of CIAS [50].

Several large systematic reviews and meta-analyses have attested the effectiveness of CR in CIAS on both cognitive and functional outcomes [55,107,108,109], as well as its good acceptability profile for participants [110] and the durability of its positive effects [111].

In particular, two large systematic reviews and meta-analyses focusing on the effectiveness of CR were recently conducted by two completely independent groups and published close to one another [55,108]. Interestingly, while the two meta-analyses followed a slightly different methodological approach as regards study inclusion criteria (one included both neurocognition- and social cognition-targeting CR programs [55], while the other focused on neurocognition-oriented interventions [108] and the performed supplementary analyses; the results were very similar, both as regards the observed effect sizes and the moderator of effects.

The presence of an active and trained therapist delivering the intervention, the structured repetition of exercises, the development of novel cognitive strategies, and the implementation of techniques to transfer cognitive gains into the real world all represent essential elements of the intervention [55,112]. Age of participants does not appear to represent a significant moderator of effects [55,113]. In this regard, CR can be offered and provide benefits in older participants, but also represents a valid strategy in a longitudinal perspective in the early-course of SSD individuals [114,115,116,117].

Considering this wealth of evidence regarding the effectiveness of CR, recent research is focusing on the implementation of this intervention in real-world rehabilitation practice. In fact, while the evidence of CR is well-attested, widespread dissemination in mental health services is lacking [56,58,118].

Recent evidence shows that CR can be easily implemented in real-world contexts even with limited available resources [119,120], while CR programs have been successfully delivered also in low-income countries such as India [121], Iran [122], Togo, and Benin [123,124].

Barriers hindering the widespread implementation of CR appear to be mostly related to the limited understanding of the benefits of this therapy on the part of mental health professionals, of public health services, of policymaker organizations, and of regulatory entities [58,118,125,126,127]. In this regard, academic and clinical partnership and collaboration, as well as advocacy from service users, families, carers, and stakeholder organizations have proven to represent powerful instruments to foster implementation [128,129].

Another category of psychosocial interventions that can provide substantial benefits in treatment of CIAS are physical exercise (PE) based interventions, also called physical activity interventions.

PE interventions, and aerobic PE in particular, are well-renowned for their positive effects on metabolic and global health-related outcomes in people with SSD [48,130,131], and have also shown reliable evidence of effectiveness on core dimensions of the disorder, such as positive and negative symptoms [132,133,134]. Several systematic reviews and meta-analysis have confirmed that PE interventions are also consistently effective in improving CIAS [132,135,136].

A more recent meta-analysis has not only confirmed this observation, but also highlighted that, similarly to CR, PE is more effective when delivered by an active and trained professional [137]. This work also observed a positive effect of a group context, as well as a dose-response effect of PE interventions, starting from an intensity of 90 min per week for a duration of at least 12 weeks. Another recent meta-analysis also confirmed that the positive effects of PE interventions are not only confined to psychopathological dimensions of SSD and CIAS but are also generalized to real-world functioning of participants [138]. Given these findings, PE can be considered an evidence based intervention to address CIAS.

In addition, CR and PE interventions can be combined into integrated rehabilitation treatment programs. According to recent evidence, the combination of CR and PE is more effective than each intervention alone and appears to provide faster improvements in CIAS, as PE appears also to enhance the cognitive effects of CR [139,140,141].

Limitations to a widespread implementation of PE interventions in rehabilitation services are akin to those observed for CR: knowledge and understanding of the benefits of this treatment, and of the importance of CIAS in the context of SSD treatment, remain limited, particularly in regulatory and policymaking contexts. Improving the awareness regarding these issues, with the aim of obtaining high level and government organizational backing as observed in some contexts [128,129], could be translated into a more consistent delivery of this treatment to people accessing mental health services with a diagnosis of SSD.

However, other strategies might further help in the implementation and delivery of effective treatments to CIAS. Moreover, novel treatments are being developed and investigated in the treatment of SSD which, based on their mechanisms of action, could provide benefits as regards CIAS.

Non-pharmacological interventions currently suggested and/or under investigation for CIAS treatment are summarized in Table 2.

## 3. Future Perspectives

Pharmacological and non-pharmacological interventions for CIAS are still in need of improvement, although evidence supporting psychosocial interventions is now solid and well proven, the diffusion of this type of intervention is yet to be improved. Furthermore, specific molecules for this crucial unresolved issue in the treatment of SSD are still in need of more evidence.

In addition, since CIAS has been proven to have a heterogeneous presentation, with different domains being affected, future efforts should be made to evaluate the efficacy of combined treatment, for instance testing a combination of non-pharmacological and pharmacological treatment. In the current literature not much evidence is available on this topic, although future studies are looking at this perspective [146,147].

Linked to the topic of the heterogeneity of clinical presentation of CIAS and of SSD in general, personalized treatments should become a focus in the future as well, concentrating on specific deficits in different domains and implementing treatments also according to the patients’ abilities, in a recovery perspective [36].

Looking at future perspectives for this field, it is necessary to consider that the development and the use of novel technologies have brought new perspectives in several fields of medicine, including psychiatry.

The COVID-19 pandemic had an enormous impact on mental health services across the globe [148,149,150,151] and on the lives of people with severe mental illness such as SSD [152,153,154,155,156,157], but the need to avoid in-person visits ushered in an increase in the development and use of telemedicine [158,159,160].

In fact, telemedicine could represent a valid instrument also in the treatment of CIAS, as modern technologies could allow an effective remote delivery of CR interventions [161].

Several feasibility studies and clinical trials attested the acceptability and the effectiveness of remotely delivered CR interventions, even before the onset of the pandemic [162,163,164,165,166,167,168].

A recent systematic review and meta-analysis confirmed that evidence-based psychosocial interventions for SSD retained their efficacy, also when remotely delivered. In particular, it was observed that CR interventions produced significant positive effects on both cognitive and functional outcomes [169]. Preliminary evidence has also been recently provided for the feasibility and efficacy of app-based and smartphone-based CR interventions [170,171], but more studies are currently required to confirm the clinical impact of this approach.

The possibility to offer remotely delivered CR interventions could substantially increase the accessibility of this type of intervention, allowing more mental service users to receive this type of treatment and contributing to wider implementation of evidence-based interventions for people with SSD.

Another instrument that has recently seen a surge in public interest and also in the attention of the scientific community thanks to current advances in the technological field is that of immersive virtual reality.

Virtual reality has been theorized as an instrument to effectively deliver treatments in an immersive manner for different mental health conditions [172,173,174]. Different systematic reviews have observed that virtual reality interventions appear to be feasible and effective in populations of individuals diagnosed with SSD [175,176], while a recent meta-analysis including 11 different studies reported that virtual reality CR interventions produced significant positive effects on CIAS [177].

However, these findings cannot be considered as conclusive for several different reasons: many studies included small populations or presented significant quality issues, an assessment of global cognitive performance was carried out in less than half of the studies, and the essential outcome of psychosocial functioning was not taken into account.

Despite these limitations, virtual reality CR interventions appear as promising instruments that could offer more immersive and more ecologically valid rehabilitation interventions for CIAS. More large and high-quality trials are currently underway to investigate the effectiveness of virtual reality CR in SSD [178,179] and also in other severe mental illnesses [180,181]: the results of these studies will provide further valuable information.

Following these results, comparing virtual reality-based CR interventions to traditional ones on outcomes of acceptability and effectiveness, also of engagement and of subjective benefits, as well as investigating the cost-effectiveness of virtual reality CR interventions represent the next steps for research in this field.

Another intervention that is currently gaining increasing attention in the treatment of SSD is non-invasive brain stimulation (NIBS). NIBS techniques aim to treat mental health conditions by modulation of brain activity through the use of magnetic or electric induction; transcranial direct current stimulation (tDCS) and transcranial magnetic stimulation (TMS) are both techniques that have been explored in the treatment of SSD and could have a positive effect on CIAS [182,183].

Both TMS [184] and tDCS [142,143,144,145] have shown consistent positive effects on CIAS, but these effects currently appear to be limited mostly to the working memory domain. This lack of evidence of generalized cognitive effects and the still limited evidence of effects on psychosocial functioning outcomes do not currently allow NIBS to be suggested as a top-tier treatment for CIAS. However, the high heterogeneity observed as regards stimulation protocol intensity, duration, and even electrode placement currently represents an important confounding factor, and more research is currently needed to assess the most effective stimulation protocol for CIAS, which could indeed represent a valid and effective treatment.

NIBS treatment can also be easily combined with CR interventions, structuring integrated protocols that could be effective on CIAS as well as in other important core features of SSD [185].

## 4. Discussion

CIAS represents one of the most prevalent, most impactful, and overall most important features of SSD [4,12,17], particularly as it remains an unaddressed issue for many individuals with SSD in real-world clinical contexts [56,58,118].

This is a particularly problematic issue as effective treatments to address CIAS have been identified and validated. These include the adaptation of pharmacological treatment [50] and the delivery of evidence-based psychosocial interventions such as CR [55,107,108] an PE [136,137,138]. Fostering the implementation of these intervention in rehabilitation practice should represent a key goal for practitioners in the perspective of providing the most effective treatments for people living with SSD.

On the other hand, novel treatments are being developed and their effectiveness on CIAS is currently being assessed. These include both new pharmacological treatments [45,82] and new technologies for delivering psychosocial interventions, such as remote delivery programs [161,169] and virtual reality [177]. NIBS techniques are also a growing field of study, and protocols and programs that could provide substantial benefits on CIAS in a reliable manner could be identified in the near future [145].

Future studies will inform the scientific community on the efficacy, the effectiveness, and also on the relationship between cost and usefulness of these new therapeutic approaches. It is probable that some, but not all, of these novel developments will be translated into valid treatments that can be effectively provided for people living with SSD. These treatments will likely be implemented alongside or in combination with interventions of currently established effectiveness.

As novel evidence emerges, scientists and clinicians should work hand in hand to ensure that the most effective treatments for people living with SSD are not only identified but also made available in real-world settings in order to provide meaningful improvements in several areas of their lives.

## Figures and Tables

**Table 1:** Pharmacological treatments for CIAS.

Molecule	Mechanism of Action	Level of Evidence
SGAs	SGAs show a lower anticholinergic burden, therefore decreasing the onset of CIAS as a treatment side-effect	Suggested by the EPA in patients experiencing CIAS due to their more favorable cognitive profile [50]
Memantine	Inhibition of acetylcholinesterase	Preliminary evidence of positive effects [83,84,85]
Galantamine	Positive allosteric modulation of alpha-7 nicotinic acetylcholine receptors	Preliminary evidence of positive effects [86]
N-Acetylcysteine	Anti-inflammatory action	Preliminary evidence of positive effects [91]
Minocycline	Anti-inflammatory action	Preliminary evidence of positive effects [90,92,93].
Iclepertin	Selective glycine transporter 1 inhibition	Preliminary evidence of positive effects reported in a Phase II placebo-controlled randomized trial [97]
luvadaxistat	D-amino acid oxidase	Preliminary evidence of positive effects reported in a randomized, placebo-controlled trial [100]
Xanomeline-trospium	A combination of muscarinic agonist acting on M1, M4, and M5 receptors (xanomeline) and a peripheral muscarinic receptor antagonist that does not cross the blood–brain barrier, reducing cholinergic adverse effects without hindering the therapeutic effects (trospium)	Preliminary evidence of positive effects reported in a Phase 3 randomized clinical trial [103]

CIAS (Cognitive impairment associated with schizophrenia); EPA (European Psychiatry Association); SGA (Second generation antipsychotic).

**Table 2:** Non-pharmacological interventions for CIAS.

Type of Intervention	Main Details	Level of Evidence
CR	the presence of an active and trained therapist delivering the intervention improves the effect the structured repetition of exercises, the development of novel cognitive strategies, and the implementation of techniques to transfer cognitive gains into the real world all represent essential elements of the intervention	Suggested by the EPA as the intervention with the highest available degree of recommendation in the EP guidance for the treatment of CIAS [50]
PE	aerobic PE appears to be the most effective treatment (compared to other types of PE) PE appears to be more effective when delivered by an active and trained professional A dose-response effect of PE interventions has been observed, starting from an intensity of 90 min per week for a duration of at least 12 weeks	Evidence of positive effects reported in several systematic reviews and meta-analysis [132,135,136]
CR + PE	According to recent evidence, the combination of CR and PE is more effective than each intervention alone and appears to provide faster improvements in CIAS, as PE appears to also enhance the cognitive effects of CR	Evidence of positive effects reported in different RCTs [139,140,141]
NIBS	Both TMS and tDCS have shown consistent positive effects on CIAS, but these effects currently appear to be limited mostly to the working memory domain	Preliminary evidence of positive effects [142,143,144,145]

CIAS (Cognitive impairment associated with schizophrenia); CR (Cognitive remediation); NIBS (Non-invasive brain stimulation); PE (Physical exercise); tDCS (Transcranial direct current stimulation); TMS (Transcranial magnetic stimulation); RCTs (Randomized controlled trials).

## Data Availability

No new data were created or analyzed in this study.
